# Possibilities and Limitations of ICP-Spectrometric Determination of the Total Content of Tin, Its Inorganic and Organic Speciations in Waters with Different Salinity Levels—Part 1: Determination of the Total Tin Content

**DOI:** 10.3390/molecules28165967

**Published:** 2023-08-09

**Authors:** Zaual Temerdashev, Pavel Abakumov, Mikhail Bolshov, Darya Abakumova, Alexander Pupyshev

**Affiliations:** 1 Analytical Chemistry Department, Faculty of Chemistry and High Technologies, Kuban State University, Krasnodar 350040, Russia; pg.abakumov@gmail.com (P.A.); abakumova.dd@gmail.com (D.A.); 2 Institute for Spectroscopy Russian Academy of Sciences, Moscow 108840, Russia; mbolshov@mail.ru; 3 Department of Physical and Chemical Methods of Analysis, Institute of Physics and Technology, Ural Federal University, Yekaterinburg 620062, Russia; pupyshev@gmail.com

**Keywords:** tin, organotin compounds, deionized and sea water, ICP-OES, ICP-MS, hydride generation, microwave digestion

## Abstract

This paper considers the features of determining the total tin content in waters with different salinity. Direct ICP-spectrometric analysis of sea waters with a salinity of more than 6‰ significantly reduced the analytical signal of tin by 70% (ICP-MS) and 30% (ICP-OES). The matrix effect of macrocomponents was eliminated by generating hydrides using 0.50 M sodium borohydride and 0.10 M hydrochloric acid. The effect of transition metals on the formation of tin hydrides was eliminated by applying L-cysteine at a concentration of 0.75 g/L. The total analyte concentrations, considering the content of organotin compounds, were determined after microwave digestion of sample with oxidizing mixtures based on nitric acid. The generation of hydrides with the ICP-spectrometric determination of tin leveled the influence of the sea water matrix and reduced its detection limit from 0.50 up to 0.05 µg/L for all digestion schemes. The developed analysis scheme made it possible to determine the total content of inorganic and organic forms of tin in sea waters. The total content of tin was determined in the waters of the Azov and Black seas at the levels of 0.17 and 0.24 µg/L, respectively.

## 1. Introduction

The natural entry of tin into waters is due to metal-containing minerals and anthropogenic sources—the production of various paints, glass products, waterproofing coatings, pesticides, antifouling agents and the metallurgical industry [1]. Organotin compounds found in waters are products of inorganic tin methylation occurring in waters and biological tissues due to biogeochemical cycles in the aquatic environment [2,3].

Organic tin compounds are the most dangerous pollutants of aquatic ecosystems among different tin compounds. The permissible daily dose of the sum of tributyltin, triphenyltin, dibutyltin and di-n-octyltin is 0.1 µg/kg of body weight expressed in terms of tin [4]. Organotin compounds (OTC) are widely used throughout the world, causing significant damage to localized coastal areas. More than 800 organotin compounds are known, which have a wide range of applications with a total production of up to 80,000 t/year [5]. Since the 1960s, OTC have been actively used as biocides in antifouling systems, aquaculture and agriculture. As a result, environmental pollution is found in water, bottom sediments and soil, as these accumulate in biota, as well as in the food chain. Marine organisms at high trophic levels are more susceptible to them, which is why the International Maritime Organization has introduced a ban on the use of tributyltin antifouling paints since September 2008 [6].

Inorganic tin compounds are considered relatively non-toxic, and their content in waters is not standardized, except for fishery waters [7]. However, the inorganic speciation of tin can be subjected to biomethylation and transform into OTC, which are characterized by higher toxicity [8].

The sea waters studied in this work are characterized by different levels of salinity. The Azov sea belongs to brackish water bodies due to the mixing of river and Black Sea waters. The salinity of the northeastern part of the sea and coastal areas is significantly influenced by the Don and Kuban rivers, and from the south there is a significant influence of the Black sea waters. The average value of the salinity of the water of the Azov sea is 6–10.5‰ [9]. The Black sea is characterized by horizontal water circulation along the entire perimeter, and the average value of the surface layer water salinity at depths up to 200 m is approximately 18‰; at levels below the two-hundred-meter mark it is 22–22.5‰ [10].

The ICP-OES and ICP-MS are more preferable for the determination of tin in natural objects due to their high sensitivity, a wide range of determined concentrations, and their ability to be used in the direct determination of the analyte. The review article [11] considers the main problems of the analysis of natural and drinking waters via the ICP-MS related to sampling, matrix effects, spectral interferences and gives recommendations for their elimination and accounting. The expediency of combining the ICP-OES and ICP-MS in the analysis of waters with high salinity to expand the list of determined elements and improve the precision of the analysis is substantiated. The use of ICP-MS in the direct determination of tin in sea waters is difficult due to the matrix effect and spectral interference [12,13]. The review article [14] discusses the spectral interference of polyatomic ions in the determination of ^120^Sn, ^104^Ru^16^O, ^104^Pd^16^O, ^108^Pd^12^C, which must be accounted for using correction equations. This problem arises when the content of palladium and ruthenium in the analyzed sea water is sufficiently high. Isobar interference to the ^120^Sn isotope is possible only from the ^120^Te isotope [15]. Considering the ratio of the considered isotopes in terms of their abundance (32.6 for ^120^Sn and 0.1 for ^120^Te) [15] and the low concentration of ^104^Ru, ^104^Pd, ^108^Pd and ^120^Te in the analyzed waters, the effect of these elements in the determination of ^120^Sn can be neglected.

The authors of [16] described a method for determining the total tin content in fresh water with low salinity. They managed to achieve a tin detection limit of 0.05 µg/L for ICP-MS in direct analysis. However, in this case, water samples were analyzed without sample preparation, which may be incorrect due to the thermal stability of organotin compounds. By introducing NaCl into calibration solutions, the authors of [17] tried to avoid the dilution of the analyzed solutions during ICP-MS analysis of waters with high salinity (up to 30 g/L NaCl); however, this procedure does not completely eliminate the matrix effect.

ICP-OES is less sensitive to matrix effects in the determination of tin compared to ICP-MS [11]. Thus, the authors of [18] determined tin in surface waters with low mineralization at the level of 10 μg/L without the stage of concentration. However, in this case, the determined concentrations of tin were rather high (about 100 µg/L). To reduce the limits of detection, methods of preliminary concentration of the analyte are used. For example, it is possible to determine tin in natural waters via ICP-OES at a level of 7 ng/L with chemical extraction preconcentration in an ultrasonic field [19], and in sea waters at a level of 8.5 µg/L using sorption preconcentration [20]. However, the preconcentration methods used in [19,20] significantly complicate the analysis. In addition, the studies [18,19,20] did not consider the possible presence of organotin compounds in the analyzed waters.

This work discusses the possibility of the determination of the total tin content in the waters of the Azov and Black seas, both via direct and hyphenated methods based on ICP-spectrometric analysis. Issues related to taking into account matrix interferences in the determination of tin via ICP-spectrometry and methods for their leveling have been considered. The results of such a study will make it possible to develop a comprehensive method for assessing the toxicological impact of tin on the aquatic ecosystem under study. The method for determining the total tin content described in this work is a reliable way to confirm the analyte content in aquatic ecosystems, without which it is incorrect to carry out further material analysis of tin forms.

## 2. Results and Discussion

### 2.1. Conditions for the ICP-Spectrometric Determination of Tin

It was experimentally found that the widest range of linearity of the analytical signal of tin (AS_Sn_) dependence on analyte concentration, and the highest sensitivity and accuracy of its determination were achieved for an emission wavelength of 189.989 nm (ICP-OES) (R^2^ = 1.000) and at the ^120^Sn isotope (ICP-MS) (R^2^ = 1.000).

**Figure 1:**
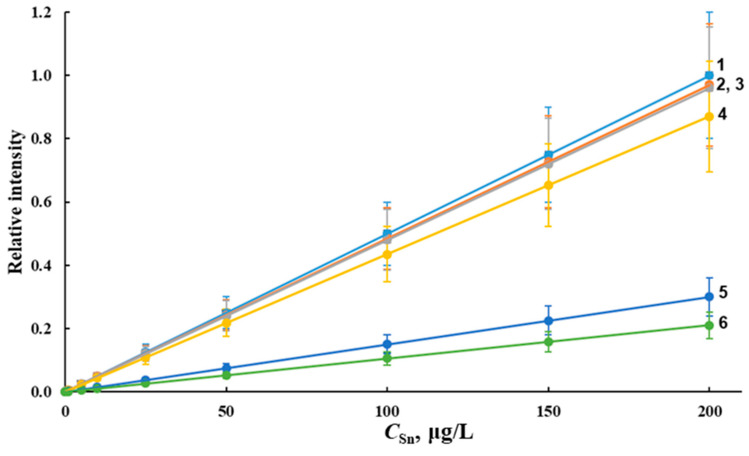
Calibration graphs for tin in solutions prepared in deionized (1), model fresh (2), model sea water with a salinity of 6‰ (5) and diluted 50 times (3), model sea water with a salinity of 18‰ (6) and diluted 100 times (4) for ICP-MS.

**Figure 2:**
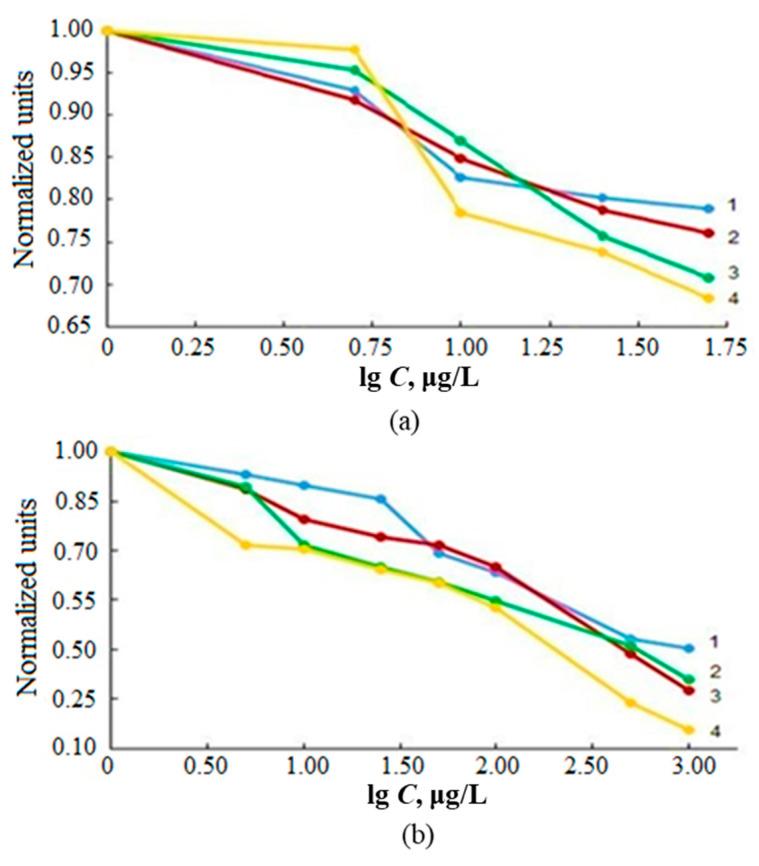
The influence of the concentration of the main anions (**a**)—PO_4_^3−^ (1), Cl^−^ (2), NO_3_^−^ (3), SO_4_^2−^ (4) and cations (**b**)—Mg^2+^ (1), Ca^2+^ (2), Na^+^ (3), K^+^ (4) of sea water on AS_Sn_ (normalized units) for ICP-MS.

**Figure 3:**
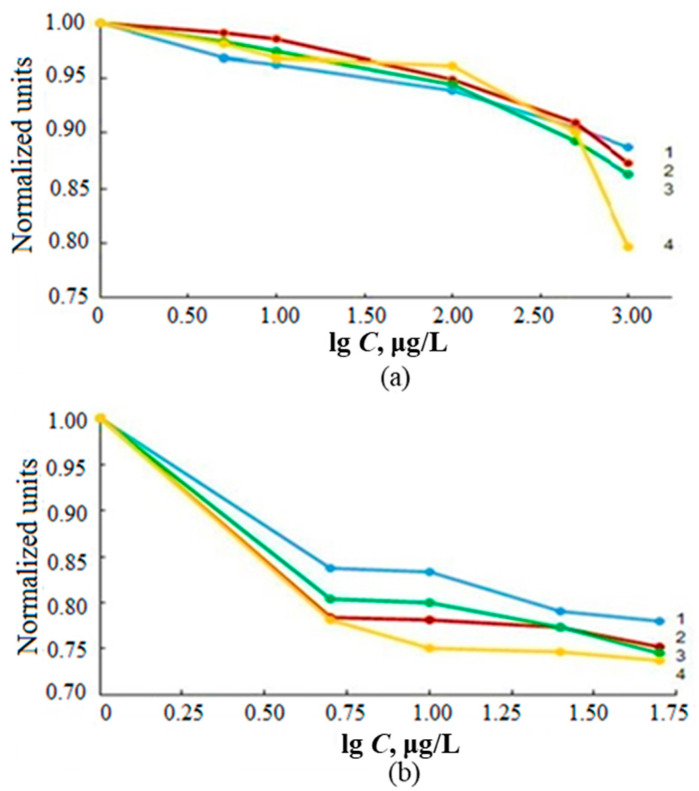
The influence of the concentration of the main anions (**a**)—PO_4_^3−^ (1), Cl^−^ (2), NO_3_^−^ (3), SO_4_^2−^ (4) and cations (**b**)—Mg^2+^ (1), Ca^2+^ (2), Na^+^ (3), K^+^ (4) of sea water on AS_Sn_ (normalized units) for ICP-OES.

The combined effect of matrix components on AS_Sn_ in ICP-OES and ICP-MS determinations was studied via the construction of the calibration graphs using reference solutions (tin concentration range 0.1–20,000 μg/L) prepared in deionized water, model fresh and sea waters with different salinity (0.5‰—fresh water; 6‰—water of the Azov sea; and 18‰—surface water of the Black sea). An analysis of the calibration graphs and approximation coefficients showed the linearity of the dependence of AS_Sn_ on the tin concentration in the range of 0.1–100 µg/L for ICP-MS and 0.5–20,000 µg/L for ICP-OES in solutions prepared in deionized water. The content of basic cations and anions in fresh waters and sea waters with low salinity is usually below 1 g/L. The effect of the main matrix components on AS_Sn_ was studied using calibration graphs based on the solutions prepared in deionized, and model fresh and sea waters (Figure 1). With an increase in the salinity of sea water, the slope of the calibration curve decreased, regardless of the detection method used. At the same time, the calibration curves constructed in model solutions of natural and deionized waters had the same slope, indicating a low content of matrix elements in these solutions and, consequently, a minimal effect of the matrix in determining tin. Figure 1 shows that the salinity of 6‰ (Figure 1, curve 5) and 18‰ (Figure 1, curve 6) significantly affect AS_Sn_ and its decrease is maximum. This fact can be fully explained by the influence of the matrix components of sea water on AS_Sn_ (Figure 2 and Figure 3).

The type of calibration curves for the ICP-OES determination of tin is approximately the same. A significant decrease in AS_Sn_ for ICP-OES (up to 40%) and ICP-MS (up to 80%) was observed in the analysis of undiluted model sea waters with a salinity of 6‰ and 18‰. The matrix effect on AS_Sn_ was significantly reduced via the dilution of the studied samples with deionized water by a factor of 100 at a salinity of 18‰ and was significantly eliminated via dilution by a factor of 50 at a salinity of 6‰ (Figure 1, curves 3 and 4).

The experimentally detected influence of the concentrations of the main anions of sea water (Cl^−^, SO_4_^2−^, NO_3_^−^ and PO_4_^3−^) on AS_Sn_ (Figure 2a) demonstrates a significant decrease in the signal even at concentrations of less than 10 mg/L for both methods.

High concentrations of sea water cations (Na^+^, K^+^, Ca^2+^ and Mg^2+^) cause a decrease in AS_Sn_, especially for the ICP-MS determination (Figure 2b). The matrix effect of seawater cations on AS_Sn_ at ICP-OES (Figure 3) is, apparently, associated with different reasons: the behavior of easily ionizable elements when the sample is introduced in the form of an aerosol into the plasma torch, and the processes of an analyte atomization and ionization in plasma, especially when the spectral lines of the analyte have different origins. In the case of ICP-MS, we also observed the effect of transport of the resulting ions through the sampler and skimmer to the ion optics of the detector.

As can be seen from the graphs in Figure 2 and Figure 3, a significant decrease in the analytical signal AS_Sn_ was observed already at low concentrations of the matrix components of sea waters. These data indicate that the direct ICP-spectrometric determination of tin in waters of different salinity requires the elimination of the influence of the matrix of the object under study. To reduce the influence of matrix effects and increase the stability of the spectrometer, the analyzed solution is usually diluted, but such a procedure for determining tin did not provide correct and reproducible results due to the low content of the analyte in sea waters.

### 2.2. Influence of Chemical Forms of Tin in Water on the Analytical Signal in ICP-Spectrometric Determination

**Figure 4:**
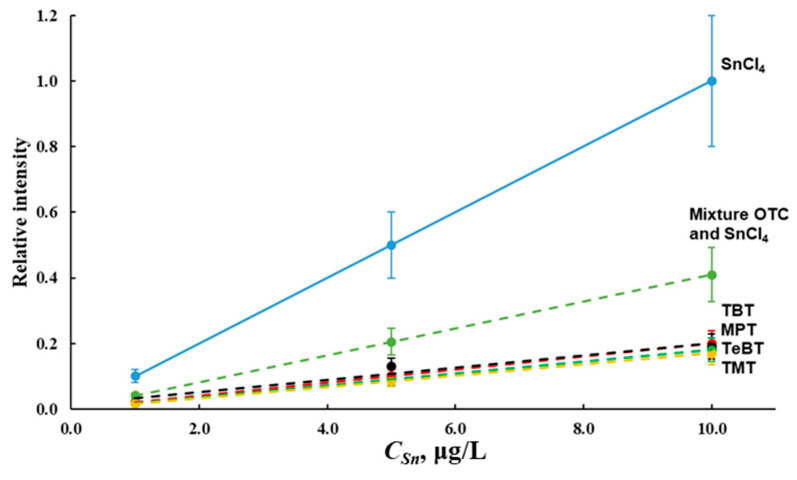
Calibration graphs for the determination of various tin compounds in deionized water by the ICP-OES without sample pretreatment.

In addition to the main components of sea water, organotin compounds also have a significant effect on AS_Sn_ in the direct ICP-spectrometric determination of tin. Figure 4 shows calibration curves for the direct determination of tin via the ICP-OES in various solutions prepared in deionized water: pure tin (IV) chloride; OTC TBT:TeBT:TMT:MPT in the ratio 1:1:1:1; mixtures of tin (IV) chloride and individual OTC with a total concentration of tin 1.00, 5.00 and 10.0 µg/L. The resulting calibration curves for the ICP-MS determination of tin in the studied concentration range look similar. The size of the additives was chosen considering the permissible concentrations of OTC in the waters [4] and the level of tin content in the waters of the Azov and Black seas.

A decrease in the analytical signal of tin by more than 80% in the analysis of waters containing OTC was observed. The same picture was obtained in the analysis of waters containing both tin (IV) chloride and OTC (Figure 4). Evidently, elimination of the influence of the organic matrix of the OTC on AS_Sn_ is possible after pretreatment of the samples with the destruction of the thermally stable matrices of the OTC.

### 2.3. Microwave Sample Pretreatment of Waters of Different Salinity for the Determination of Total Tin

To account for the influence of the organic matrix of the OTC on AS_Sn_ and to determine the total tin content, the optimal conditions for the digestion of waters containing OTC were established by taking into account the data of [21,22,23]. The influence of OTC on AS_Sn_ was eliminated using various oxidizing mixtures based on nitric acid, including mixtures of nitric acid with H_2_O_2_ and HCl (Table 1). The efficiency of the oxidizing agents was estimated via the analysis of model waters of various salinities containing a mixture of OTC with the addition of 5.00 μg/L (1.25 μg/L each of TBT, TeBT, TMT and MPT) in terms of inorganic tin. Tin content was controlled via ICP-spectrometry (Table 1). The volumes of additives were chosen considering the permissible concentrations of OTC in waters [4]. The results of the determination were evaluated according to the standard deviation [24] and the value of the quality of the obtained results (test recovery) [25].

Optimal digestion of waters was achieved (with an acceptance criterion of 95% < R < 105%) using nitric acid as an oxidizing agent. Schemes of microwave digestion with oxidizing agents 4.0 mL HNO_3_ + 1.0 mL HCl as well as 3.0 mL HNO_3_ + 2.0 mL H_2_O_2_ gave satisfactory results with an acceptance criterion of 90% < R < 110% and can be used to prepare samples for analysis.

**Figure 5:**
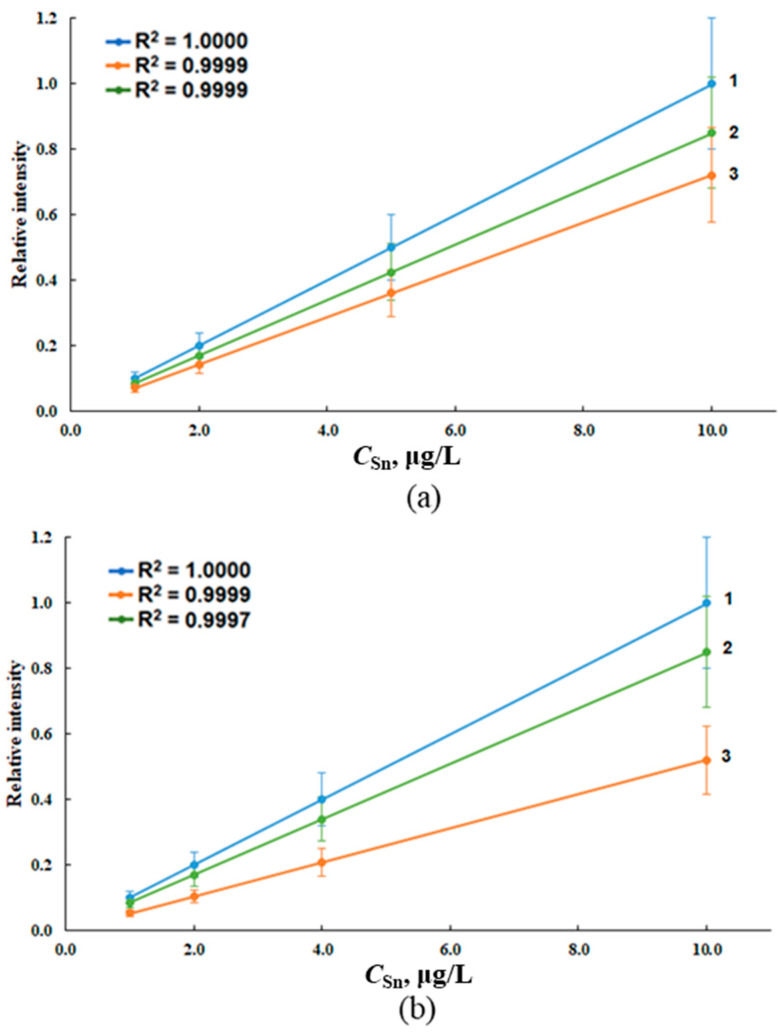
Calibration curves for the determination of tin in deionized water (1), model sea waters with salinity of 6‰ (2) and 18‰ (3) after microwave digestion via ICP-OES (**a**) and ICP-MS (**b**).

The total concentration of tin after microwave digestion was determined from calibration curves constructed on model water samples of various salinities containing OTC and tin (IV) chloride with tin concentrations in the ranges of 1.00–10.0 μg/L (ICP-OES) and 0.50–5.0 μg/L (ICP-MS) (Figure 5).

The proportional growth of AS_Sn_ with an increase in the concentration of tin confirms the completeness of the decomposition of OTC in waters with different salinities. At the same time, we note that the calibration graphs retain the slopes characteristic of waters with different salinity (Figure 5); therefore, when determining tin in various types of water, calibration graphs must be constructed based on the corresponding model sea waters.

### 2.4. Hydride Generation as a Method of Concentration and Determination of Tin

The hydride generation of tin prior to ICP-spectrometric determination can reduce the limits of analyte quantification compared to direct analysis by increasing the efficiency of a sample introduction into argon plasma and by minimizing the matrix effect [26]. However, the correct determination of tin in waters with different salinities using the hydride generation technique is possible only after optimizing the conditions of sample pretreatment considering the tin inorganic and organic forms in the analyzed solution.

#### 2.4.1. Study of the Conditions for the Hydride Generation of Tin

**Figure 6:**
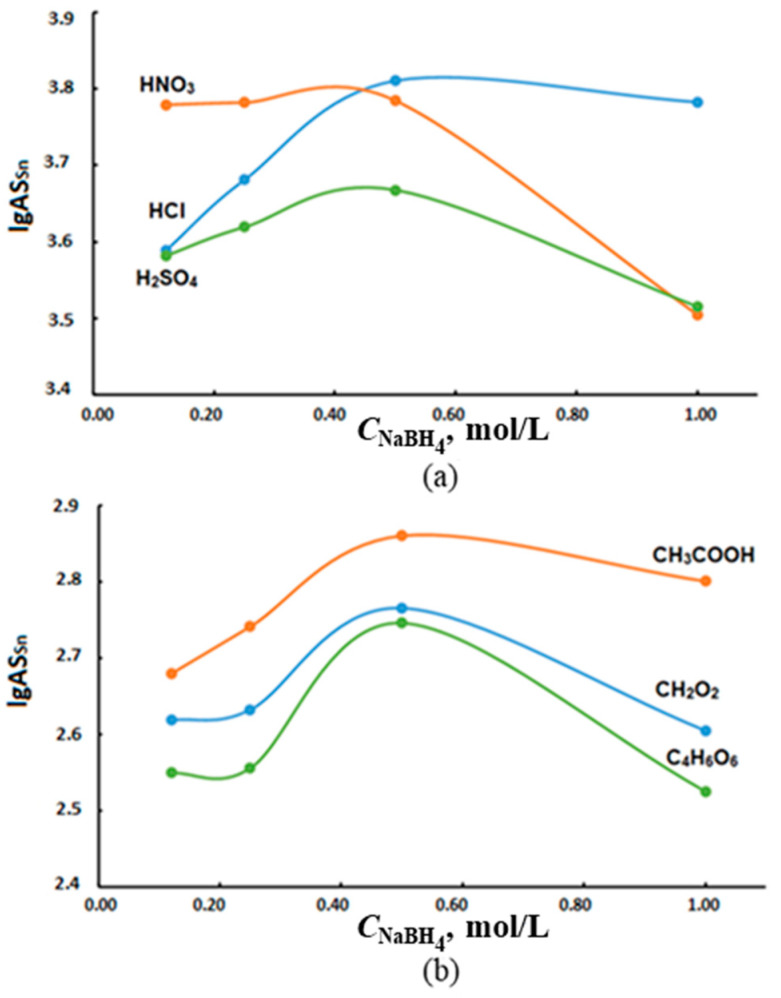
The effect of 0.10 mol/L inorganic (**a**) and 3.00 mol/L organic (**b**) oxidizing agents on AS_Sn_ during the generation of tin hydrides with a solution of sodium tetraborate.

**Figure 7:**
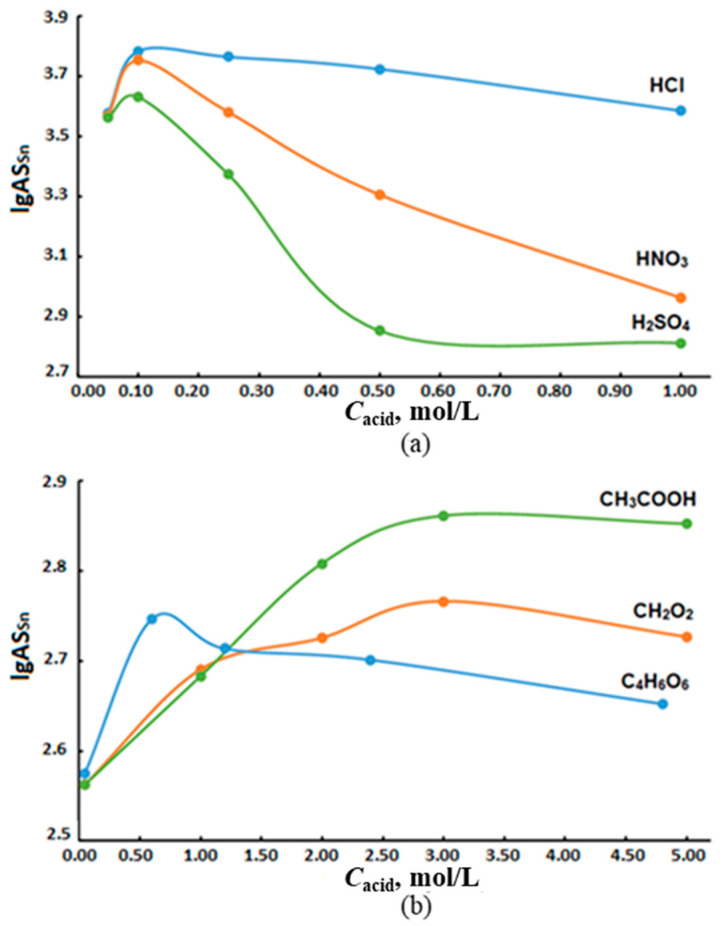
Influence of the concentrations of (**a**) inorganic and (**b**) organic oxidants on AS_Sn_ during the generation of tin hydrides with a 0.5 mol/L solution of sodium tetraborate.

The main factors affecting the efficiency of tin hydrides generation are the concentration of the reducing agent, the choice of the oxidizing agent and its concentration, which determine the rate and completeness of the reaction. The hydride generation of tin was carried out using sodium borohydride and the following oxidizing agents: hydrochloric, nitric, sulfuric, formic, acetic and tartaric acids. The optimization of the conditions for hydride generation of tin was studied within the reduction agent content range of 0.12–1.00 mol/L at constant concentrations of oxidizing agents: mineral (0.10 mol/L) and organic (3.00 mol/L) acids (Figure 6 and Figure 7). The concentration and selection of oxidants were optimized considering the literature [27,28,29,30,31] and experimental data.

Following, the solutions of individual compounds and their mixtures in deionized water were analyzed. The results are as follows: tin (IV) chloride, TBT, TeBT, TMT and MPT; mixtures of tin (IV) chloride with OTC (TBT, TeBT, TMT and MPT) with concentrations 2.00 µg/L of each analyte in terms of inorganic tin. The volumes of the additions were selected corresponding to the tin content in the waters of the Azov and Black seas. Low concentrations of nitric acid as an oxidizing agent provide high values for AS_Sn_, which decreases at NaBH_4_ concentrations above 0.5 mol/L (Figure 6a). The authors of [32] do not recommend the use of nitric acid as an oxidizing agent, since its active interaction with NaBH_4_ leads to the creation of an acidic environment in the reaction cell and a decrease in the reduction of tin, which affects the stability of AS_Sn_. The replacement of AS_Sn_ with sulfuric acid as an oxidizing agent apparently also increases the acidity of the reaction mixture and complicates the determination of tin hydrides. Organic acids (formic, acetic and tartaric) used in the hydride generation showed weak acidic properties (lg AS_Sn_ did not exceed 2.83), which indicated a low efficiency of their use (Figure 6b).

From the graphs in Figure 6, it can be seen that the ~0.5 mol/L solution of NaBH_4_ is optimal and provides a maximum tin signal using any organic or inorganic acid as an oxidizing agent, of which only HCl did not have a pronounced maximum in the range of concentrations used (Figure 7).

The obtained results show that the optimal generation of tin hydrides occurs for 0.50 mol/L NaBH_4_ solution and 0.10 mol/L HCl solution. The use of a reductant with a higher concentration disrupts the stability of the hydride system, leading to plasma disruption.

#### 2.4.2. Influence of Organotin Compounds in Water on the Hydride Generation of Tin

Considering the factor of the possible influence of OTC on the generation of tin hydrides with subsequent ICP-spectrometric determination, we studied the conditions for analyzing water samples with and without the use of microwave digestion.

Initially, we analyzed solutions prepared on deionized water with individual compounds of tin (IV) chloride, TBT, TeBT, TMT and MPT with concentrations of each analyte of 0.10, 0.50, 1.00, and 5.00 μg/L in terms of inorganic tin. The obtained calibration curves with the generation of hydrides are similar to the curves for direct ICP-spectrometric determination (Figure 4). We also analyzed solutions prepared on deionized water containing a mixture of tin (IV) chloride with TBT, TeBT, TMT and MPT in an equimolar ratio with a total analyte concentration of 0.10, 0.50, 1.00, and 5.00 μg/L with and without the use of microwave digestion. Data (Table 2) showed that it is difficult to correctly determine the total tin content in waters containing OTC via the generation of hydrides without preliminary sample preparation, which ensured the destruction of thermally stable OTC matrices.

On the other hand, the generation of tin hydrides after microwave digestion without the re-solution of the mineralizate is difficult because high residual content of oxidants in the mineralizate (about 10% vol) resulted in poor reproducibility because of plasma instability up to its disruption. A significant decrease in AS_Sn_ due to high concentrations of acids, including nitric acid, confirms this assumption (Figure 7a). The excess content of oxidizing agents was eliminated by evaporating the mineralizate to wet salts and their re-dissolution in deionized water followed by ICP-spectrometry analysis (Table 2).

The introduction of an additional stage of sample pretreatment made it possible to determine the total content of tin via the generation of hydrides in waters below 0.10 µg/L by both ICP-spectrometric methods.

#### 2.4.3. Influence of Water Salinity on the Determination of Tin via Hydride Generation

The isolation of hydride-forming analytes from the analyzed solution makes it possible to minimize matrix interference from the majority of elements that do not form stable volatile compounds [33]. The influence of the main macro components of sea waters on AS_Sn_ was studied using model solutions of waters of different salinity within the analyte concentration range of 0.05–1.00 μg/L. Microwave digestion of the samples destroyed the thermally stable OTC, thereby eliminating the possibility of their influence on the conditions for the hydride generation of tin. The conditions for determining tin via the proposed schemes of sample pretreatment did not change with an increase in water salinity. This result confirms the possibility of eliminating interference from the matrix components during the hydride generation of tin. Therefore, the salinity of sea water can be ignored and calibration curves for tin determination using the hydride generation technique can be constructed basing on the tin solutions in deionized water.

#### 2.4.4. Effect of Transition Metals on the Determination of Tin Hydrides

During the chemical generation of tin hydrides, transition metals in water samples such as Ni, Co, Cu, Fe, etc. can cause interferences [29,33,34]. Interference from these metals is associated with a competitive interaction with NaBH_4_, as well as the catalytic effect on the decomposition of tin hydrides by the reduced interfering metal [29].

**Figure 8:**
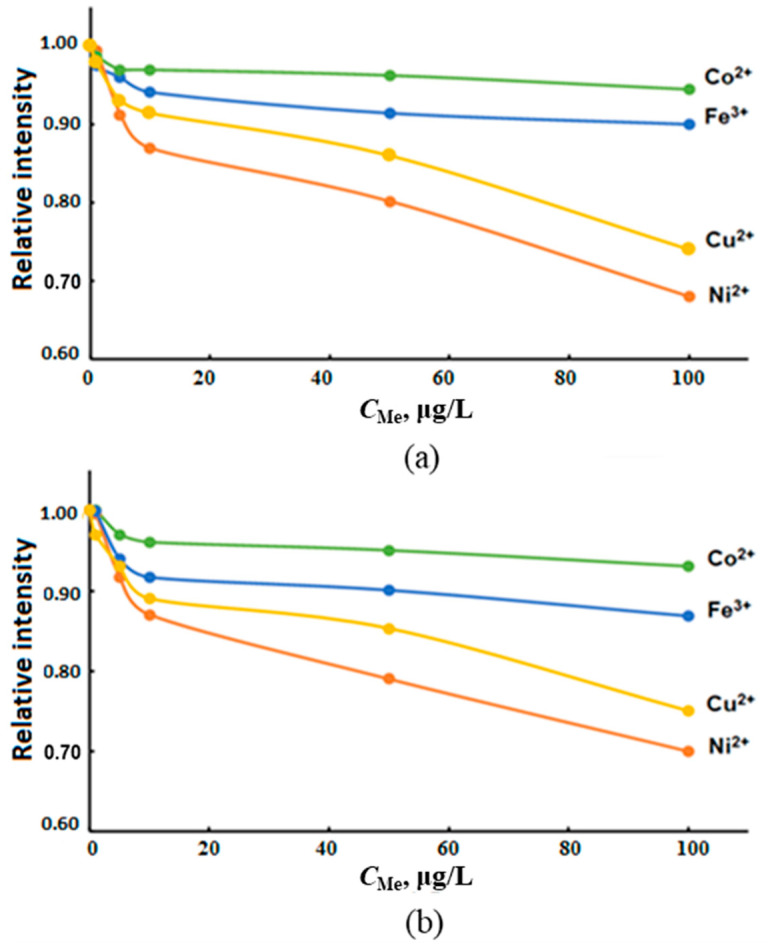
Dependence of AS_Sn_ of tin hydrides on the concentration of transition metals in solutions of deionized water with a metal concentration of 1.00 μg/L ICP-OES (**a**) and ICP-MS (**b**).

**Figure 9:**
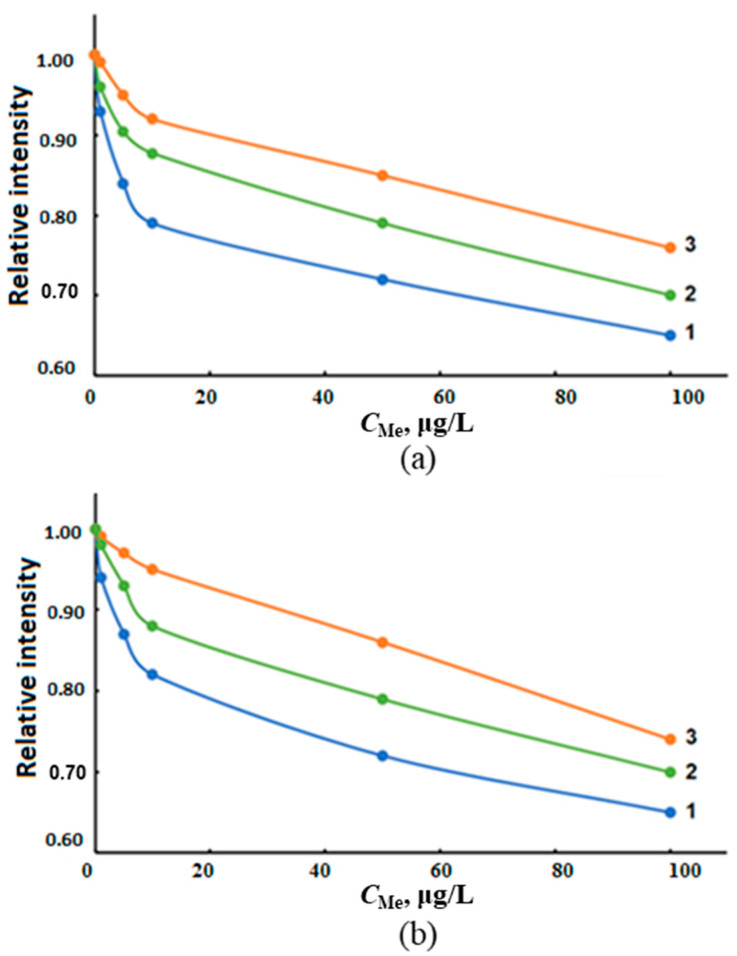
The influence of the total content of Ni^2+^, Co^2+^, Cu^2+^ and Fe^3+^ with the concentration of each in the range of 1.0–100.0 µg/L in solutions based on deionized (1) and model waters with a salinity of 6‰ (2) and 18‰ (3) by ICP-OES (**a**) and ICP-MS (**b**) determination of tin hydrides.

The influence of transition metals on the determination of tin hydrides was studied by measuring the dependence of AS_Sn_ on the concentrations of Ni^2+^, Co^2+^, Cu^2+^ and Fe^3+^ varied in the range of 1.00–100 μg/L in solutions of deionized water containing 1.00 μg/L inorganic tin (Figure 8). We also studied the change in AS_Sn_ from the sum of transition metals in model solutions of various salinities containing interferents in a ratio of 1:1:1:1 with a total metal content of 1.00, 5.00, 10.0, 50.0 and 100 μg/L (Figure 9).

The greatest decrease in AS_Sn_ during ICP-OES and ICP-MS determination was observed in the presence of Ni^2+^ and Cu^2+^ (up to 30%), and Fe^3+^, Co^2+^—up to 10% (Figure 8). The cumulative effect of the total content of interferents practically did not depend on the salinity of the samples and caused the decrease in AS_Sn_ by about 30% (Figure 9). In order to eliminate the effect of transition metals on AS_Sn_, we studied the possibility of using masking agents in the determination of inorganic tin in sea waters.

When determining hydride-forming elements in the presence of transition metals, masking agents were used, which increase the efficiency of hydride formation by reducing the analyte to a more reactive form or by interacting with an interfering agent [29,30,35]. L-cysteine, EDTA, C_4_H_6_O_6_, KI, and CH_4_N_2_S reduce the possibility of hydride formation of a competing reaction and contribute to the correct determination of tin hydrides [35] due to the binding of transition metals.

The effectiveness of masking agents In the determination of tin hydrides was evaluated via the analysis of the solutions of deionized water containing 1.00 μg/L of inorganic tin and transition metals Ni^2+^, Co^2+^, Cu^2+^ and Fe^3+^ with a concentration of 20.0 μg/L each (Table 3). In this case, some masking agents were introduced into the analyzed samples (L-cysteine, tartaric acid and EDTA) to exclude the possible binding of the analyte into complex compounds, and others into NaBH_4_ solutions (potassium iodide and thiocarbamide). Considering the experimental and published data [29,35], the exposure ranges for the concentrations of masking agents were determined, which were as follows: 0.50–2.50 mg/L for EDTA; 0.50–1.25 g/L for L-cysteine; 1.00–4.00 g/L for C_4_H_6_O_6_; 0.05–1.00 g/L for KI and 0.50–1.25 g/L for CH_4_N_2_S. Experimental errors were evaluated via the standard deviation [24] and the test recovery [25] acceptance criterion.

The depressing effect of Ni^2+^, Co^2+^, Cu^2+^ and Fe^3+^ was maximally eliminated with L-cysteine at a concentration of 0.75 g/L with an acceptance criterion of 95% < R < 105%. The active form of formation of stable tin hydrides manifests itself at the oxidation state Sn^4+^ [36]. L-cysteine, in addition to eliminating chemical interference from transition metals, is able to modify the NaBH_4_–Sn reaction system due to the formation of reactive tin complexes and stabilization of the analyte solution, which increases the efficiency of stannane formation and increases AS_Sn_ [35,37]. The mechanism of action of other masking agents is associated with the reduction of hydride-forming elements to a more reactive form.

**Figure 10:**
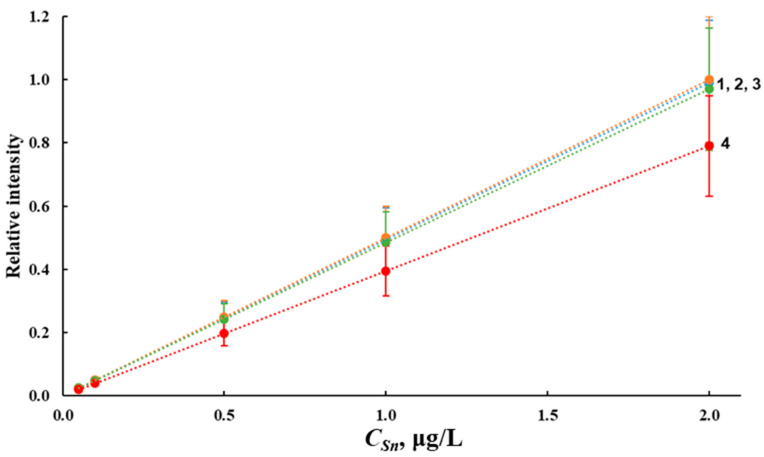
Calibration curves for ICP-OES determination of tin hydrides in solutions prepared on deionized water with tin (IV) chloride (1); L-cysteine and tin (IV) chloride (2); L-cysteine, tin (IV) chloride and transition metals (3); tin (IV) chloride and transition metals (4).

Calibration curves for the determination of tin hydrides by ICP-OES and ICP-MS without and with the use of L-cysteine were constructed using the solutions of tin in deionized water within the concentration range of 0.05–2.00 μg/L and transition metals Ni^2+^, Co^2+^, Cu^2+^, Fe^3+^ and L-cysteine with a concentration of 20.0 μg/L each (Figure 10). Figure 10 presents the calibration curves for the ICP-OES determination of tin hydrides; they are similar for the ICP-MS determination.

The slope of the calibration curve for the hydride generation of tin in the absence of a masking agent (Figure 10, curve 4) indicates the need to account for the effect of transition metals. The introduction of L-cysteine into the analyzed solution eliminated the effect of transition metals on the results of tin determination (Figure 10, curves 1, 2 and 3).

The generation of hydrides under optimized conditions enabled the determination of the inorganic form of tin in the range of 0.05–2.0 μg/L via ICP-OES and 0.03–2.0 μg/L via ICP-MS without additional stages of analyte preconcentration and accounting for the matrix composition of waters. The use of L-cysteine had a positive effect both on the masking of transition metals and on the efficiency of the hydride generation of tin.

### 2.5. Limits of Quantification of Tin and Analysis of Real Seawater Samples

Under optimal conditions for determining the total content of the analyte, the limits for the determination of tin in stabilized 1% HCl model solutions prepared in deionized, model fresh and sea waters with different salinities were established (Table 4).

The use of the hydride generation technique in combination with the ICP-spectrometric analyte determination practically reduced the effect of the water matrix and enabled us to obtain the values of the limits of tin quantification, independently of the salinity of the samples, at the level of 0.03 and 0.05 μg/L for the ICP-MS and ICP-OES determination of the analyte, respectively.

A satisfactory repeatability of the results of determining the total tin content was observed after microwave digestion of waters using direct sample introduction in the range of 0.45–10.0 μg/L (ICP-OES), 0.40–5.0 μg/L (ICP-MS) and using generation of hydrides in the ranges of 0.05–2.0 µg/L (ICP-OES) and 0.03–2.0 µg/L (ICP-MS).

The developed technique was used to determine the total content of inorganic and organic tin in water samples of the Azov and Black seas (Table 5). The accuracy of tin determination was controlled via the standard addition technique. Mixture of tin (IV) chloride and OTC in stoichiometric ratios was added to the analyzed real sea water samples with a total analyte content of 1.00 μg/L for direct determination and 0.10 μg/L for hydride generation.

The sensitivity of ICP-spectrometric analysis was insufficient for the determination of the total content of the analyte with direct injection of the sample in plasma. Total average tin content in the waters of the Azov and Black seas of 0.17 and 0.24 μg/L, respectively, was measured via the developed complex technique of hydrides’ generation after microwave digestion of the samples.

## 3. Materials and Methods

### 3.1. Research Objects

Samples of natural waters taken from the surface layer of the Azov and Black seas were chosen as objects. The sampling and storage were carried out in polypropylene containers, considering the recommendations [11]. To exclude the ingress of suspended particles, the samples were filtered through a paper filter “blue” tape (pore size of 3–5 μm). Selected sea water probes were stabilized using hydrochloric acid at pH = 2, which is universal for fixing all speciations of tin [38]. After sampling and preservation, water can be stored in a refrigerator at 4 °C for analysis up to 15 days [38]. Model water samples with salinities of 6 and 18‰ were prepared in deionized water using reagents of reactive purity, considering the recommendations [39]. The choice of salinity of the model water samples corresponds to the salinity of the waters of the Azov [9] and Black [10] seas.

In fresh and sea water, inorganic tin is present in the tetravalent form [40]. When studying the influence of the chemical matrix on the determination of tin, we were also guided by the fact that the chemical composition and ratios of the main macrocomponents in sea waters in all regions of the globe are equal in accordance with the Marcet principle [41]. Model sea waters with the chemical composition, ratios of the main macro components and salinity of the Azov and Black seas were prepared considering the data of [9,10,41] for an adequate assessment of their influence on the determination of the chemical speciations of tin.

### 3.2. Reagents

For experimental studies, high-purity argon (99.998%) was used; 15.4 mol/L HNO_3_, 12 mol/L HCl, 10 mol/L H_2_O_2_, tributyltin chloride (TBT, 98%), trimethyltin chloride (TMT, 98%), monophenyltin trichloride (MPT, 98%) and tetrabutyltin (TeBT, 94%) were purchased from Sigma-Aldrich (St. Louis, MO, USA); sodium borohydride (NaBH_4_, 96%) was purchased from PanReac AppliChem (Council Bluffs, IA, USA); sodium chloride (NaCl, 99.9%), magnesium chloride 6-water (MgCl_2_·6H_2_O, 98.0%), sodium sulfate anhydrous (Na_2_SO_4_, 99.5%), anhydrous calcium chloride (CaCl_2_, 96.5%), potassium chloride (KCl, 99.8%), sodium carbonate acid (NaHCO_3_, 99.8%), boric acid (H_3_BO_3_, 99.9%) were used.

All solutions were prepared in deionized water with a maximum resistivity of 18.2 MΩ cm^−1^ obtained in a sub-boiling distillation unit DuoPUR Subboiling Distilling System (Milestone, Milan, Italy). Calibration solutions in the range of 0.05–20,000 μg/L for ICP-OES and 0.01–100 μg/L for ICP-MS were prepared using a stock tin solution (C_Sn_ = 1 g/L) purchased from Inorganic Ventures (Christiansburg, VA, USA) with 1% HCl.

The influence of matrix effects and ways to eliminate them in the determination of tin were studied using stock solutions of sodium, potassium, calcium, magnesium, sulfate ions, nitrate ions, phosphate ions with a concentration of 1 g/L and chloride ions with a concentration of 10 g/L purchased from Inorganic Ventures (USA). The model seawater solution consisted of 22.00 g of NaCl, 9.70 g of MgCl_2_·6H_2_O, 3.70 g of Na_2_SO_4_, 1.00 g of CaCl_2_, 0.65 g of KCl, 0.20 g of NaHCO_3_ and 0.023 g of H_3_BO_3_ dissolved in 1 L deionized water.

Tin was determined via hydride generation using 0.50 mol/L sodium borohydride solution stabilized with a 0.10 mol/L sodium hydroxide solution. The reducing agent solution was prepared on the day of analysis by dissolving a sodium borohydride (m = 1.90 g) in 100 mL of deionized water. Solutions of oxidizing agents–15.4 mol/L HNO_3_, 12 mol/L HCl, 17.9 mol/L H_2_SO_4_, 26.4 mol/L formic (CH_2_O_2_), 17.4 mol/L acetic (C_2_H_4_O_2_) and tartaric acid (C_4_H_6_O_6_, 99.9%) were prepared by diluting acids with deionized water to the required concentrations in the ranges of 0.05–1.00 mol/L for mineral and 0.05–5.00 mol/L for organic acids.

The influence of transition metals on the determination of tin in waters was evaluated using stock solutions of iron (III), nickel (II), cobalt, copper (II) (1 g/L) purchased from Inorganic Ventures (USA). Potassium iodide (99.5%), thiocarbamide (99.0%), L-cysteine hydrochloride (98.0%), tartaric acid (99.9%) and ethylenediaminetetraacetic acid (EDTA, 99.8%) were used as masking agents for transition metal.

The modeling of samples containing OTC in deionized water was carried out considering their possible content at the level of maximum permissible concentrations in the water areas of the studied aquatic ecosystems.

### 3.3. Instrumentation

Inductively coupled plasma mass-spectrometer iCAP RQ (Thermo Scientific, Waltham, MA, USA) and inductively coupled plasma optical emission spectrometer iCAP-7400 series (Thermo Scientific, Waltham, MA, USA) were used in these experiments. The operating conditions of the devices, considering the specifics of the analyzed object, were studied using a MicroMist concentric nebulizer purchased from Glass Expansion (Melbourne, Australia).

#### 3.3.1. Optimization of Operating Modes of Spectrometers

Operating parameters of the ICP-MS and ICP-OES spectrometers: the rates of the cooling, auxiliary, and nebulizer argon flows, and the power of the RF-generator were optimized for realization of the best sensitivity, reproducibility, and accuracy of tin determination in water. The above parameters were optimized by analyzing solutions with a constant tin concentration prepared in deionized, model sea and fresh waters. The selected operating parameters of the spectrometers are summarized in Table 6.

For a chemical hydride generation system, the Integrated Hydride Kit for ICP-systems of the Thermo Fisher Scientific (Waltham, MA, USA) was used. In an acrylic reaction cell filled with glass beads to increase the reaction yield, tin hydrides were formed by mixing the reagents and the acidified sample solution introduced into the hydride system in parallel. The resulting volatile compounds were transported to the plasma torch of the spectrometer by an argon flow through a membrane filter with a Teflon surface, which served as a gas–liquid separator.

#### 3.3.2. Influence of Matrix Components of Sea Waters on the Determination of Tin

Since the matrix composition of sea waters, in contrast to that of fresh waters, was stable [42,43,44], the influence of the main macro components of sea water, Na^+^, K^+^, Ca^2+^, Mg^2+^, Cl^−^, SO_4_^2−^, NO_3_^−^ and PO_4_^3−^, on the AS_Sn_ was studied. We also considered the influence of OTC on the direct ICP-spectrometric determination of tin without any sample pretreatment. For this, tin (IV) chloride and OTC (TBT, TeBT, TMT and MPT) were sequentially diluted with deionized water to concentrations of 1.00, 5.00 and 10.0 μg/L each in terms of tin. The size of the additives was chosen considering the permissible concentrations of OTC in the waters [4] and the level of tin content in the waters of the Azov and Black seas.

#### 3.3.3. Microwave Sample Pretreatment of Water of Different Salinity

In the ICP analysis of waters containing tin (IV) chloride and OTC, one can expect a significant underestimation of AS_Sn_ [16]. To a certain extent, this fact can be explained by the high thermal stability of the OTC [45,46,47]. For example, tributyltin chloride boils without decomposition above 170 °C [45], trimethyl tin chloride boils at 154 °C [46] and tetrabutyl tin chloride boils at 145 °C [47]. The thermal stability of tin (IV) chloride (boils at 114.15 °C) [48] does not affect the determination of inorganic tin in the ICP analysis of natural waters with low salinity [16].

To assess the effect of inorganic and organic forms of tin in their ICP-spectrometric determination, we studied the effect of OTC on AS_Sn_ in water with and without the use of sample pretreatment. To detect the total tin content in the waters, the OTC was converted into an inorganic form via microwave digestion using the microwave system MARS 6 (CEM, Charlotte, NC, USA), considering the data [21,22] and the recommendations of the microwave system producers [23].

#### 3.3.4. Conditions for the Generation of Tin Hydrides

To increase the sensitivity of the determination of tin and minimize the matrix effect of the chemical composition of various types of water, the generation of analyte hydrides was used. To obtain the maximum ratio AS_Sn_ to the background signal, the operating parameters of the spectrometers were optimized for the case of hydride generation (Table 7). The hydrides were generated using a reducing agent, sodium borohydride NaBH_4_ and oxidizing agents—hydrochloric, nitric, sulfuric, formic, acetic and tartaric acids. The optimal operating parameters of the spectrometers, considering the recommendations of the hydride system producers, were established by analyzing the tin (IV) chloride solution with a concentration of 50.0 µg/L acidified by 2% hydrochloric acid [49]. The concentration of the reducing agent NaBH_4_ was varied in the range of 0.12–1.00 mol/L; the concentration of oxidizing agents was 0.10 mol/L mineral or 3.00 mol/L organic acids. The concentrations of oxidizing agents were chosen by taking into account the literature [32,33,34] and our experimental data.

#### 3.3.5. Limit of Quantification

The limits of the quantification of inorganic tin (LOQ) were determined using the calibration graphs based on reference solutions prepared in deionized water and model sea waters of different salinities. For measuring the mean level and standard deviation of blank, repeated measurements (n = 15) of blank solutions with corresponding salinity were carried out. LOQ was obtained using solutions containing tin (IV) chloride. The tin LOQ was calculated as follows [25]:

LOQ = 10 × S/b,

where S is the standard deviation of blank values at a confidence level *p* = 0.95; b is the tangent of the slope of the calibration graphs.

## 4. Conclusions and Future Perspectives

The described investigations allowed us to establish the features of the direct ICP-spectrometric determination of the total tin content in saline waters of the Azov and Black seas in the presence of organotin compounds. When determining inorganic and organo-tin compounds via ICP-spectrometry with direct sample injection in plasma, it was necessary to overcome the influence of water salinity and the presence of OTC in the analyzed waters on the results of the analysis. Before detecting an analyte in water containing organotin compounds, microwave digestion of the analyzed samples is required to convert them into the inorganic forms of tin. The highest efficiency of water samples’ pretreatment was achieved with microwave digestion using nitric acid as an oxidizing agent. To increase the sensitivity of the analysis and for the compensate matrix effect associated with water salinity, the tin hydride generation technique was proposed.

Under optimized conditions for water analysis via ICP-spectrometry with hydride generation, the limits of quantification of tin were found, which were 0.05 μg/L for ICP-OES and 0.03 μg/L for ICP-MS regardless of the water salinity level. The developed methods were tested on model water solutions with different salinities, as well as on samples of the waters of the Azov and Black seas; the total tin contents in the latter were 0.17 and 0.24 µg/L, respectively.

It can be assumed that the use of ICP-OES and ICP-MS in the analysis of waters with different salinity levels in combination with hydride generation enables researchers to solve the problem of determining total tin content. The problem of the separate determination of inorganic and organic tin compounds remains unresolved, which, apparently, will be successfully solved after the preliminary separation of the compounds.

## Figures and Tables

**Table 1:** Efficiency of microwave digestion schemes of water samples of different salinity with 5.00 µg/L mixtures OTC (by 1.25 μg/L TBT, TeBT, TMT and MPT) in terms of tin (*n* = 3, *p* = 0.95).

Type of Water	The Composition of the Oxidizer	Found, in Terms of Tin, μg/L		Recovery, % (Acceptance Criteria: 95% < R < 105%)	
		ICP-OES	ICP-MS	ICP-OES	ICP-MS
**Deionized water**	Without oxidizer	2.2 ± 0.3	1.5 ± 0.3	44	30
	1.2 mL HNO_3_ + 0.6 mL H_2_O_2_	3.9 ± 0.6	3.4 ± 0.5	78	68
	5.0 mL HNO_3_	4.9 ± 0.8	5.1 ± 0.7	98	101
	4.0 mL HNO_3_ + 1.0 mL HCl	4.8 ± 0.7	4.9 ± 0.7	95	98
	2.5 mL HNO_3_ + 2.5 mL HCl	4.8 ± 0.7	4.2 ± 0.6	96	84
	3.0 mL HNO_3_ + 2.0 mL H_2_O_2_	5.4 ± 0.8	5.3 ± 0.8	108	106
	1.0 mL HNO_3_ + 4.0 mL HCl	5.1 ± 0.7	3.6 ± 0.5	101	72
**Model water with a salinity of 6‰**	Without oxidizer	2.2 ± 0.3	1.5 ± 0.2	44	29
	1.2 mL HNO_3_ + 0.6 mL H_2_O_2_	3.8 ± 0.6	3.3 ± 0.5	75	67
	5.0 mL HNO_3_	4.7 ± 0.7	4.9 ± 0.7	95	97
	4.0 mL HNO_3_ + 1.0 mL HCl	4.7 ± 0.7	4.7 ± 0.7	94	94
	2.5 mL HNO_3_ + 2.5 mL HCl	4.4 ± 0.7	4.2 ± 0.6	89	85
	3.0 mL HNO_3_ + 2.0 mL H_2_O_2_	4.9 ± 0.7	4.9 ± 0.7	98	98
	1.0 mL HNO_3_ + 4.0 mL HCl	4.8 ± 0.7	4.7 ± 0.7	97	94
**Model water with a salinity of 18‰**	Without oxidizer	2.3 ± 0.4	1.7 ± 0.3	46	35
	1.2 mL HNO_3_ + 0.6 mL H_2_O_2_	3.6 ± 0.5	3.7 ± 0.6	71	75
	5.0 mL HNO_3_	4.8 ± 0.7	4.8 ± 0.7	97	96
	4.0 mL HNO_3_ + 1.0 mL HCl	4.8 ± 0.7	4.8 ± 0.7	95	96
	2.5 mL HNO_3_ + 2.5 mL HCl	4.6 ± 0.7	4.7 ± 0.7	93	94
	3.0 mL HNO_3_ + 2.0 mL H_2_O_2_	4.9 ± 0.7	4.9 ± 0.7	98	99
	1.0 mL HNO_3_ + 4.0 mL HCl	4.4 ± 0.7	4.5 ± 0.7	88	90

**Table 2:** ICP-spectrometric determination of tin in water via hydride generation without and with microwave digestion (*n* = 3, *p* = 0.95).

Analyte	Added Total Tin Concentration, μg/L	Found, μg/L		Recovery, % (Acceptance Criteria: 95% < R < 105%)	
		ICP-OES	ICP-MS	ICP-OES	ICP-MS
**Tin without microwave digestion determined**	0.10	<0.05	<0.02	–	–
	0.50	0.17 ± 0.03	0.10 ± 0.02	34	20
	1.00	0.26 ± 0.05	0.19 ± 0.04	26	19
	5.00	1.3 ± 0.3	1.0 ± 0.2	26	20
**Inorganic tin determined after mineralizate redissolution**	0.10	0.09 ± 0.02	0.11 ± 0.02	90	91
	0.50	0.5 ± 0.1	0.5 ± 0.1	94	108
	1.00	1.0 ± 0.2	1.0 ± 0.2	99	105
	5.00	5 ± 1	5 ± 1	100	101
**Total content of tin after microwave digestion and redissolution of mineralizate**	0.10	0.10 ± 0.02	0.11 ± 0.02	100	110
	0.50	0.5 ± 0.1	0.4 ± 0.1	94	88
	1.00	1.0 ± 0.2	0.9 ± 0.2	98	90
	5.00	5 ± 1	5 ± 1	99	102

**Table 3:** Efficiency of masking agents in solutions on deionized water containing 1.00 μg/L of inorganic tin and Ni^2+^, Co^2+^, Cu^2+^ and Fe^3+^ with concentrations of 20.0 μg/L each in determining tin hydrides (*n* = 3, *p* = 0.95).

Masking Agent	Concentration of Masking Agent, g/L	Found Concentration of Tin, μg/L		Recovery, % (Acceptance Criteria: 95% < R < 105%)	
		ICP-OES	ICP-MS	ICP-OES	ICP-MS
**EDTA**	0.0005	0.4 ± 0.1	0.5 ± 0.1	40	50
	0.0010	0.6 ± 0.1	0.6 ± 0.1	60	60
	0.0015	0.5 ± 0.1	0.6 ± 0.1	50	60
	0.0020	0.5 ± 0.1	0.5 ± 0.1	50	50
**L-cysteine**	0.50	0.9 ± 0.2	0.9 ± 0.2	90	90
	0.75	1.0 ± 0.2	1.0 ± 0.2	100	100
	1.00	0.9 ± 0.2	0.9 ± 0.2	90	94
	1.25	0.8 ± 0.2	0.9 ± 0.2	80	90
**C_4_H_6_O_6_**	1.00	0.6 ± 0.1	0.6 ± 0.1	60	60
	2.00	0.6 ± 0.1	0.6 ± 0.1	60	60
	3.00	0.6 ± 0.1	0.6 ± 0.1	60	60
	4.00	0.5 ± 0.1	0.5 ± 0.1	50	50
**KI**	0.05	0.5 ± 0.1	0.5 ± 0.1	50	50
	0.10	0.5 ± 0.1	0.5 ± 0.1	50	50
	0.50	0.5 ± 0.1	0.5 ± 0.1	50	50
	1.00	0.5 ± 0.1	0.4 ± 0.1	50	40
**CH_4_N_2_S**	0.50	0.5 ± 0.1	0.5 ± 0.1	50	50
	0.75	0.9 ± 0.2	0.8 ± 0.2	90	80
	1.00	0.7 ± 0.2	0.7 ± 0.2	70	70
	1.25	0.6 ± 0.1	0.6 ± 0.1	60	60

**Table 4:** Limits of tin quantification in model waters of different salinity.

Object of Study	Limits of Quantification, μg/L			
	Direct Injection		Hydride Generation	
	ICP-OES	ICP-MS	ICP-OES	ICP-MS
**Deionized water**	0.32	0.03	0.05	0.03
**Model water with a salinity of 6‰**	0.40	0.37	0.05	0.03
**Model water with a salinity of 18‰**	0.47	0.45	0.05	0.03

**Table 5:** Total tin content in water samples of the Azov and Black seas (*n* = 3, *p* = 0.95).

Sample Injection	Water Sample	Sample Analyte Concentration, μg/L		Introduced, μg/L		Found, μg/L		Recovery, %	
		ICP-OES	ICP-MS	ICP-OES	ICP-MS	ICP-OES	ICP-MS	ICP-OES	ICP-MS
**direct injection**	Azov sea	<0.40	<0.37	1.00		1.2 ± 0.2	1.2 ± 0.2	100	99
	Black sea	<0.47	<0.45			1.2 ± 0.2	1.3 ± 0.2	97	98
**hydride generation**	Azov sea	0.17 ± 0.03	0.16 ± 0.03	0.10		0.26 ± 0.05	0.27 ± 0.05	90	91
	Black sea	0.25 ± 0.05	0.23 ± 0.05			0.35 ± 0.07	0.34 ± 0.07	100	91

**Table 6:** Optimum operating parameters for spectrometers in the determination of tin in waters.

Parameter		iCAP RQ Mass Spectrometer	iCAP-7400 Spectrometer (Axial Overview of Plasma)
**Analyte**		^120^Sn	Sn II 189.989 nm
**Applied power, W**		1300	1150
**Argon flowrate, L/min**	Plasma-forming (cooling)	15	12
	Auxiliary	0.80	0.50
	Nebulizer	1.10	0.50
**Peristaltic pump speed, rpm**		40	50
**Sample rate, mL/min**		0.4	

**Table 7:** Optimum operating parameters for spectrometers under conditions of hydrides generation of tin.

Parameter		iCAP RQ Mass Spectrometer	iCAP-7400 Spectrometer (Axial Overview of Plasma)
**Analyte**		^120^Sn	Sn II 189.989 nm
**Applied power, W**		1300	1150
**Argon flowrate, L/min**	Plasma-forming (cooling)	15	12
	Auxiliary	0.80	0.50
	Nebulizer	0.45	
**Peristaltic pump speed, rpm**		60	30
**Sample injection**		Hydride system: oxidizer—0.10 mol/L HCl; reducing agent—0.50 mol/L NaBH_4_	

## Data Availability

Data included in article/referenced in article.

## Sample Availability

Not available.
